# New Onset of Neuro-Sjögren's Syndrome Nine Months After the Third COVID-19 Vaccine Dose: A Case Report

**DOI:** 10.7759/cureus.69562

**Published:** 2024-09-16

**Authors:** Raja Bakhsh, Khaled Dairi, Elaf Almadabgy, Amani Albiladi, Lamyaa Gamal, Duaa Almatrafi, Fatmah AlShariff, Afnan Alsefri

**Affiliations:** 1 Internal Medicine, King Faisal Hospital, Ministry of Health, Makkah, SAU; 2 Internal Medicine, Ibn Sina College, Jeddah, SAU

**Keywords:** autoimmunity, central nervous system, neuro-sjögren's syndrome, sars-cov-2, vaccination

## Abstract

Sjögren's syndrome (SS) is an autoimmune chronic inflammatory disease causing peripheral nerve system and central nervous system issues. It results from lymphocytic infiltrates in exocrine glands, coexists with rheumatoid arthritis, and manifests independently. The COVID-19 vaccine has been linked to increased autoimmune diseases and rheumatological flare-ups, possibly due to its use of adjuvants and molecular mimicry, particularly those containing aluminum. Additionally, SS-like manifestations have been reported after the infection or vaccination, potentially leading to long-term salivary secretory dysfunction. Multiple studies have suggested the presence of a special relationship between COVID-19 vaccination/infection and the emergence of autoimmune syndromes as a negative side effect of the vaccine or direct complication from infection. The time frame for the appearance of the symptoms after vaccination or disease is not well established. Some studies suggested increased risk shortly after vaccination, while others suggested a long-term association. In this case, report, and review article, we discuss the presence of a possible association with the emergence of neuro-SS in a young lady nine months after she received her third dose of COVID-19 vaccination. Furthermore, we reviewed studies highlighting the special link and relationship between the COVID-19 vaccine/infection and SS*.*

## Introduction

Sjögren's syndrome (SS) is an autoimmune chronic inflammatory illness that may cause peripheral nerve system (PNS) and central nervous system (CNS) issues. It is distinguished by the existence of lymphocytic infiltrates in the exocrine glands, primarily the lacrimal and salivary glands, which results in dry mucosal surfaces (mouth, nose, throat, eyes, and vagina) [1].

Secondary SS coexists with rheumatoid arthritis, while primary SS manifests independently. Neuropathy, a condition causing severe pain and a decline in quality of life, can also be classified [1].

The COVID-19 vaccine has been linked to an increase in autoimmune diseases and rheumatologically flare-ups, possibly due to the use of adjuvants and molecular mimicry, particularly those containing aluminum, which is more prevalent in HLA DRB1 and HLA DQB1 genes [2]. Various autoimmune disorders, including myocarditis, vaccine-induced immune thrombotic thrombocytopenia (VITT), IgA vasculitis, and systemic lupus erythematosus [3], are reported in the literature as potential negative side effects of the vaccination. SS-like manifestations have been reported after the COVID-19 infection or vaccination.

The theory behind these manifestations can be attributed to the SAR-CoV-2 virus' affinity for salivary glands, which could potentially lead to long-term salivary secretory dysfunction in 40% of survivors [4].

We aimed from this study to understand more about the special link between COVID-19 vaccination and the emergence of SS-like manifestations after getting the vaccine.

## Case presentation

A 34-year-old Saudi female, a mother of two kids, presented to the emergency department of a tertiary care hospital in Mecca City complaining of progressive numbness in both lower limbs up to the umbilicus level. She was admitted under the care of the neurology team for further investigations. She had difficulty walking three weeks before admission. Afterwards, the numbness progressed towards the upper right limb and right face, along with heaviness in the right upper limb, associated with a sudden blurry vision of the right eye. In addition, the patient exhibited urinary retention and loss of sensation over the saddle area. She reported no history of genitourinary infection, gastroenteritis symptoms, or COVID-19 infection before the onset of symptoms.

Upon physical examination and review of history, it was observed that the patient had dry eyes and mouth, no history of other rheumatological features, and no family history of connective tissue diseases. She said that her symptoms began nine months after her booster dose of the COVID-19 mRNA Moderna vaccine. Neurological examination showed preserved power in both upper limbs and a slight decline of power in both lower limbs (4 out of 5), hyperreflexia in lower limbs, and a positive right plantar reflex, while the left plantar reflex was equivocal.

Furthermore, there was hypoesthesia in both lower limbs with sensory level at D10 plus hypoesthesia of the right upper limb till mid-arm and right side of face and neck. Lab results are shown in Table 1 and Table 2.

**Table 1:** CBC and renal panel results CBC: complete blood count; WBC: white blood cell; HGB: hemoglobin; MCV: mean corpuscular volume; NEUT. count: neutrophil count; LYMP. count: lymphocyte count; MONO. count: monocyte count; BASO. count: basophil count; BUN: blood urea nitrogen; CRE2: creatinine

	Ranges	Reference ranges
CBC		
WBC count	10.43×10^3^/UL	4,000-11,000 cells/µL
HGB	12.9 g/dL	Men: 13.8-17.2 g/dL
		Women: 12.1-15.1 g/dL
MCV	64.8 FL	80-100 fL
Platelet count	479×10^9^/L	150,000-450,000 platelets/µL
NEUT. count	7.25×10^3^/UL	1,500-8,000 cells/µL
LYMP. count	2.58×10^3^/UL	1,000-4,800 cells/µL
MONO. count	0.27×10^3^/UL	100-700 cells/µL
BASO. count	0.04×10^3^/UL	0-300 cells/µL
Renal panel		
Sodium	134 mmol/L	135-145 mEq/L
Potassium	4.27 mmol/L	3.5-5.1 mEq/L
Chloride	105 mmol/L	98-107 mEq/L
BUN	5.03 mmol/L	7-20 mg/dL
CRE2	44.4 umol/L	Men: 0.74-1.35 mg/dL
		Women: 0.59-1.04 mg/dL

**Table 2:** Liver panel and thyroid function results ALT: alanine aminotransferase; AST: aspartate aminotransferase; TSH: thyroid-stimulating hormone; FT4: free thyroxine; FT3: free triiodothyronine

	Results	Reference ranges
Liver panel		
ALT	12 U/L	7-56 U/L
AST	19 U/L	10-40 U/L
Albumin	29 g/L	3.5-5.0 g/dL
Bilirubin (total)	2 umol/L	0.1-1.2 mg/dL
Alkaline phosphatase	52 U/L	44-147 U/L
Thyroid function		
TSH	2.38 mIU/L	0.4-4.0 mIU/L
FT4	0.65 ng/dL	0.8-2.0 ng/dL
FT3	2.01 ng/L	2.0-4.4 pg/mL

The inflammatory markers erythrocyte sedimentation rate (ESR) and C-reactive protein (CRP) were 36 mm/hour and 2.08 mg/dL, respectively. Chest X-ray results showed no abnormality, and the patient's echocardiography was normal. However, lumbar puncture and autoimmune profile were evaluated, as shown in Table 3 and Table 4.

**Table 3:** Results of lumbar puncture CSF: cerebrospinal fluid; RR: reference range

Lumbar puncture	
Appearance	Clear
Cell count and differential	
White blood cell count	0.014×10^3^/uL
Red blood cell count	0.01×10^6^/uL
Neutrophil percentage	70%
Lymphocyte percentage	20%
Monocyte percentage	10%
CSF culture	No growth in 48 hours
CSF microbiology	No organism seen
Cytology	Non-diagnostic, virtually acellular smears
CSF chemistry	Protein: 224 mg/L; RR: 150-450 mg/L
	Glucose: 4.02 mmol/L; RR: 2.2-3.9 mmol/L

**Table 4:** Autoimmune profile

Autoimmune profile	
Aquaporin 4 Ab	Negative
ANA IF	Positive 1:320 titer with speckled pattern
Anti-Scl-70	Negative
Anti-Jo-1 Abs	Negative
Anti-dsDNA Ab	Negative
Anti-SS-B Ab	Negative
Anti-SS-A Ab	Positive
Anti-RNP AB	Negative

A nerve conduction study (NCS) was done twice, two weeks apart, in both lower and upper limbs and showed no abnormalities. However, visual acuity observed in the right eye was 6/15 and in the left eye was 6/6, with impaired color vision in the right eye and normal color vision in the left eye. Additionally, the macular function of the right eye was faulty and normal in the left eye; both eyes' anterior segment and fundus examination were normal.

MRI of the brain and spinal cord

**Figure 1:**
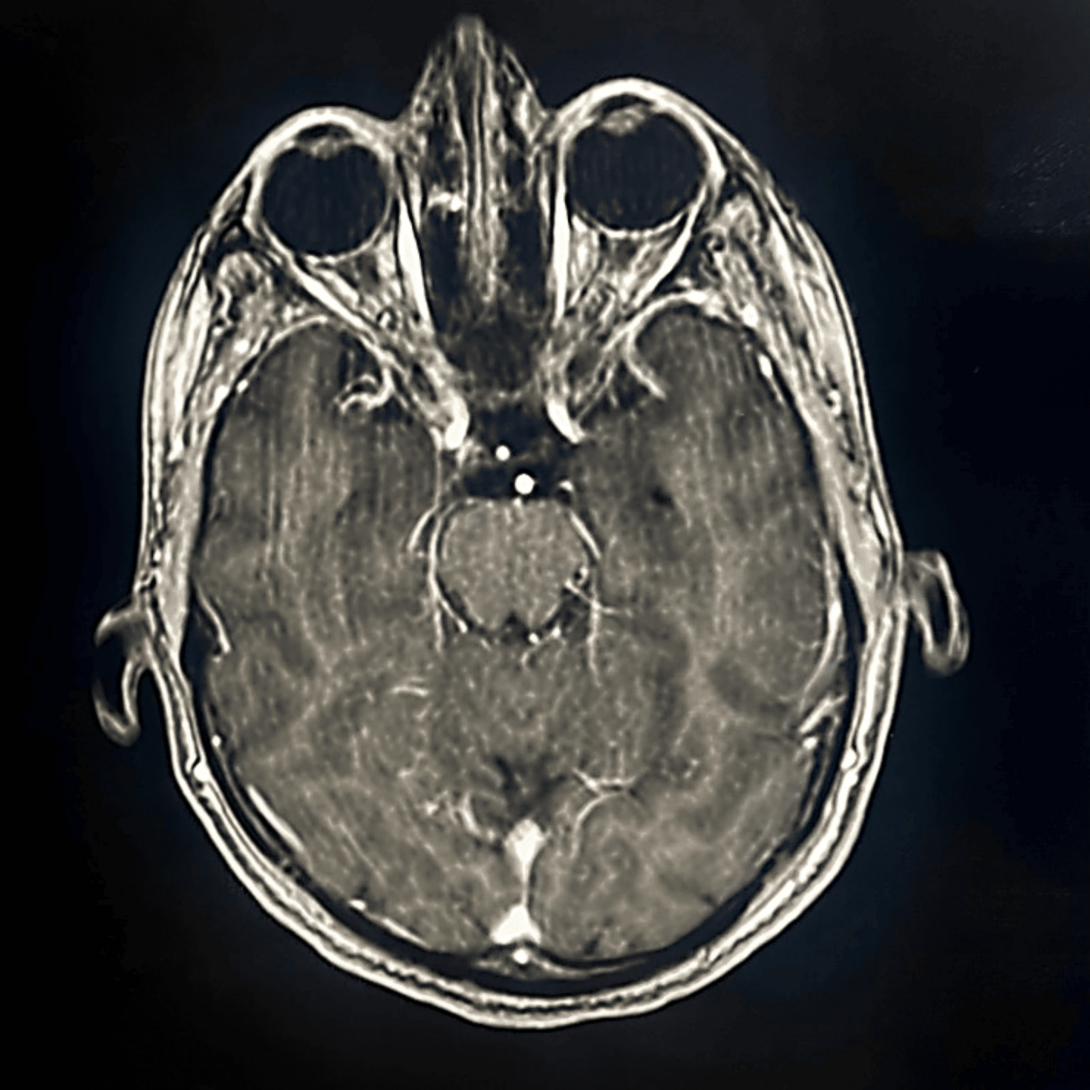
Brain MRI Both optic nerves (yellow arrows) demonstrate slight inhomogeneity and mildly increased signal intensity on both sides.

Figure 1 demonstrates the cerebral hemispheres' normal appearance and signal intensity with preserved gray-white matter differentiation, no mass effect or midline shift, normal supra- and infratentorial ventricular system, and normal appearance and signal intensity of the cerebellum. The corticomedullary junction demonstrates a focal right posterior lesion that appears as a bright signal on the FLAIR and T2 and an ISO/low signal on T1. The lesion demonstrates enhancement in the post-IV gadolinium images. The optic nerves demonstrate slight inhomogeneity and mild increased signal intensity on both sides with subtle differences in thickness.

**Figure 2:**
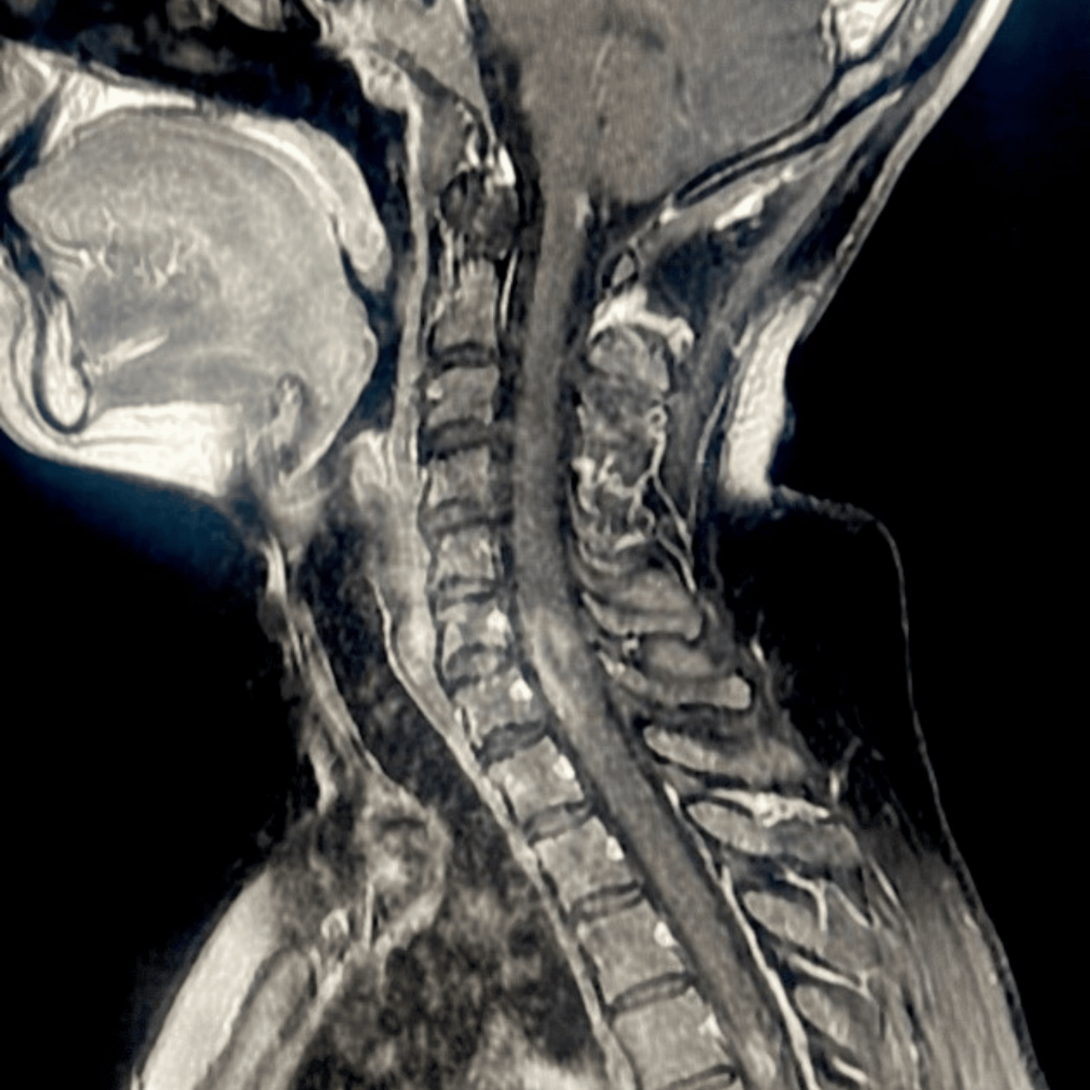
MRI of the spinal cord There is a focal right posterior lesion in the corticomedullary junction, involving short-segment lesions at the cervical and upper dorsal spinal cord levels.

The post-IV gadolinium images demonstrate enhancement on both sides, with the optic chiasm appearing within normal limits, normal enhancement of the meninges and venous dural sinuses with no gross abnormality, and good enhancement of the cerebral arteries with no gross or occlusion or aneurysmal dilation. In addition to the lesions seen in the corticomedullary junction mentioned above, other short-segment lesions may involve part of or both sides of the spinal cord, particularly in the cervical and upper dorsal regions, specifically at the C4, C5, C6, C7, and D4 vertebral body levels. These lesions show a bright signal on the STIR and T2 and appear ISO/low signal on T1 with enhancement in the post-IV gadolinium images (Figure 2).

According to the history, examination, laboratory results, and MRI, the patient was diagnosed with primary neuro-SS with CNS involvement. The management plan included the following: pulse steroid 1 gram IV methylprednisolone for five days, prednisolone 1 mg/kg (60 mg) orally daily for one month and then slowly tapering, intravenous immunoglobulin (IVIG) 0.4 mg/kg for five days, hydroxychloroquine 200 mg orally twice daily, and trimethoprim-sulfamethoxazole 960 mg orally three times per week for *Pneumocystis jirovecii* prophylaxis. On February 21, 2023, cyclophosphamide (Euro-Lupus) protocol was given 500 mg every two weeks for six doses, and mycophenolate mofetil 500 mg was given twice daily orally.

On July 19, 2023, as the patient had urticarial vasculitis proven by skin biopsy, most likely secondary to cyclophosphamide, rituximab (lymphoma dose) was initiated at 375 mg/body surface area (BSA) weekly for four doses, and the patient was maintained on rituximab 1000 mg two doses two weeks apart every six months. On December 12, 2023, as she started to complain of new lower-limb numbness, she received IVIG monthly for four months at a dose of 1 g/kg for two days. After that, the patient gradually improved, and the last neurological examination was normal.

## Discussion

Autoimmune syndromes have been observed after COVID-19 vaccines, but the exact time frame and data on immune hyperactivation post-vaccination are limited. Only a minority develop autoimmunity, supporting the idea of a genetic predisposition for vaccine-induced autoimmunity. The vaccine may cause a hyper-inflammatory state, oxidative stress, DNA damage, and autoantibodies [5].

VITT occurrences are presumably caused by platelet factor 4 antibody-mediated platelet activation via interactions between IgG and FcγR [6,7]. Vaccine adjuvants enhance vaccine immunogenicity by activating the NLRP3 inflammasome, a crucial immune system component linked to autoimmune diseases like ankylosing spondylitis, rheumatoid arthritis, systemic lupus erythematosus, SS, and systemic sclerosis [8,9].

The present case report is about a 34-year-old woman who presented with progressive numbness, difficulty in walking, and blurred vision following the COVID-19 vaccine booster, but she never had COVID-19 or any infection. Abnormal neurological and autoimmunity investigations included high ANA titers and SS-A antibodies, thus indicating primary neuro-SS manifesting with CNS involvement, as illustrated in MRI results. While zipper and other vaccinations cause autoimmune diseases, i.e., Sjögren-like syndromes, their results are not the same as infections caused by viruses since the mechanism of the vaccine can sometimes evoke hyper-inflammatory processes in genetically stimulated individuals. This immune activation is not caused by the conventional adjuvants but rather by the body's response to the presence of the intramuscularly injected mRNA complexed with lipid nanoparticles. 

The patient reported gradual improvement after several treatments, including IVIG, cyclophosphamide, and rituximab. Regarding the specific time frame of symptom onset and the pathogenesis behind the state of autoimmunity after COVID-19 vaccination, only limited data is available.

A single-center study in Saudi Arabia reported a case of SS after the second dose of the vaccine [10]. Another case series identified seven acute demyelinating diseases of the CNS, particularly those occurring just after a single or second dose [11]. It was suggested as short-term CNS demyelination/inflammation in some people after vaccination. Another case report proposes a long-term association between the development of SS and one year after receiving the COVID-19 vaccine [12]. However, autoimmunity only develops in a few cases after vaccination, suggesting a possible genetic predisposition. Furthermore, animal studies indicated that SARS-CoV-2 could be an environmental trigger for SS [13].

## Conclusions

The COVID-19 vaccines have been linked to the onset of autoimmune conditions, particularly neuro-SS, in genetically predisposed individuals. This case highlights the need for increased vigilance and research into vaccine-induced autoimmunity. The link underscores the importance of ongoing post-vaccination monitoring and understanding individual risk factors. Future research should focus on identifying genetic and immunological markers that predispose individuals to these adverse effects and evaluating vaccine safety profiles.
